# Isosorbide and dimethyl carbonate: a green match

**DOI:** 10.3762/bjoc.12.218

**Published:** 2016-10-26

**Authors:** Fabio Aricò, Pietro Tundo

**Affiliations:** 1 Department of Environmental Sciences, Informatics and Statistics, Ca’ Foscari University, Scientific Campus Via Torino 155 , 30170 Venezia Mestre, Italy

**Keywords:** carbohydrate chemistry, D-sorbitol, dimethyl carbonate, green chemistry, isosorbide

## Abstract

In this review the reactivity of the bio-based platform compounds D-sorbitol and isosorbide with green reagents and solvent dimethyl carbonate (DMC) is reported. Dehydration of D-sorbitol via DMC in the presence of catalytic amounts of base is an efficient and viable process for the preparation of the industrially relevant anhydro sugar isosorbide. This procedure is “chlorine-free”, one-pot, environmental friendly and high yielding. The reactivity of isosorbide with DMC is equally interesting as it can lead to the formation of dicarboxymethyl isosorbide, a potential monomer for isosorbide-based polycarbonate, and dimethyl isosorbide, a high boiling green solvent. The peculiar reactivity of isosorbide and the non-toxic properties of DMC represent indeed a green match leading to several industrial appealing potential applications.

## Review

### Introduction

In the last twenty years biorefinery has gained exceptional attention in the scientific community. This interest has been prompted by the substitution of petroleum-based compounds with renewable substances with the aim of establishing a bio-based economically self-sustained industry [1].

**Figure 1:**
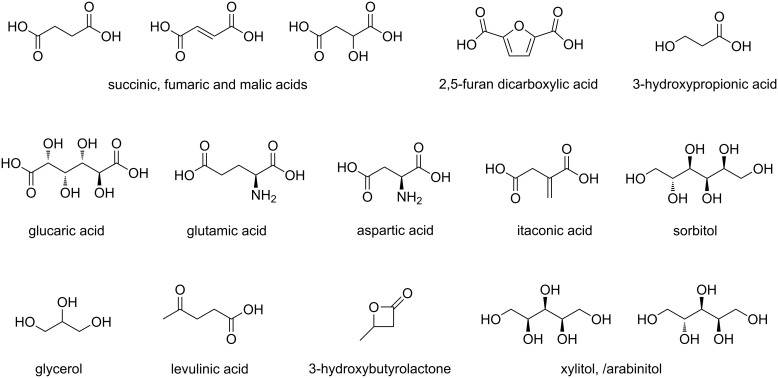
The DOE “Top 10” report [2].

In this prospect the US Department of Energy (DOE) has published a list of 15 target molecules [2], starting from 300 original candidates, that were considered of special interest for biorefinery development (Figure 1) [3]. These compounds have been selected by taking into consideration numerous factors such as available processes, economics, industrial viability, size of markets and their possible employment as a platform for the production of derivatives.

Over the years, due to the considerable progress in biorefinery development, this list, as well as the criteria used to identify bio-based products have been revised (Table 1) [1]. Several new compounds substituted the ones that have not received a great research interest. However, among the original selected chemicals, D-sorbitol, together with ethanol and glycerol, still occupy top positions as they encompass all of the desired criteria for bio-based platform compounds.

**Table 1:** Top chemical opportunities from biorefinery carbohydrates and criteria of selection.^a^

#	Bio-based compounds	Criteria

1	Ethanol	1, 2, 3, 4, 5, 6, 7, 8, 9
2	Furans	1, 2, 7, 8, 9
3	Glycerol and derivatives	1, 2, 3, 4, 5, 6, 7, 8, 9
4	Biohydrocarbons	Isoprene: 1, 2, 3, 4, 6, 7
5	Lactic acid	1, 2, 4, 7
6	Succinic acid	1, 2, 5, 6
7	Hydroxypropionic acid/aldehyde	1, 3, 4, 5
8	Levulinic acid	1, 2, 3, 5, 6, 8
9	D-sorbitol	1, 2, 3, 4, 5, 6, 7, 8, 9
10	Xylitol	1, 2, 5, 8, 9

^a^Criteria of selection: 1. The compound/technology has received significant attention in the literature. 2. The compound illustrates a broad technology applicable to multiple products. 3. The technology provides direct substitutes for existing petrochemicals. 4. The technology is applicable to high volume products. 5. A compound exhibits strong potential as a platform. 6. Scale-up of the product/technology to pilot, demo, or full scale is underway. 7. The bio-based compound is an existing commercial product, prepared at intermediate or commodity levels. 8. The compound may serve as a primary building block of the biorefinery. 9. Commercial production of the compound from renewable carbon is well established.

D-Sorbitol, namely 1,4:3,6-dianhydro-D-glucitol, is a sugar alcohol, found in nature as the sweet constituent of many berries and fruits from which it was isolated for the first time in 1872. Its large scale manufacture began in the 1950s, due to the growing applications as humectant in cosmetology and sugar substitute in confectionery. Nowadays the global market of D-sorbitol is estimated around 800 kt, half of which is produced in China with a demand currently growing at 2–3% rate annually.

**Figure 2:**
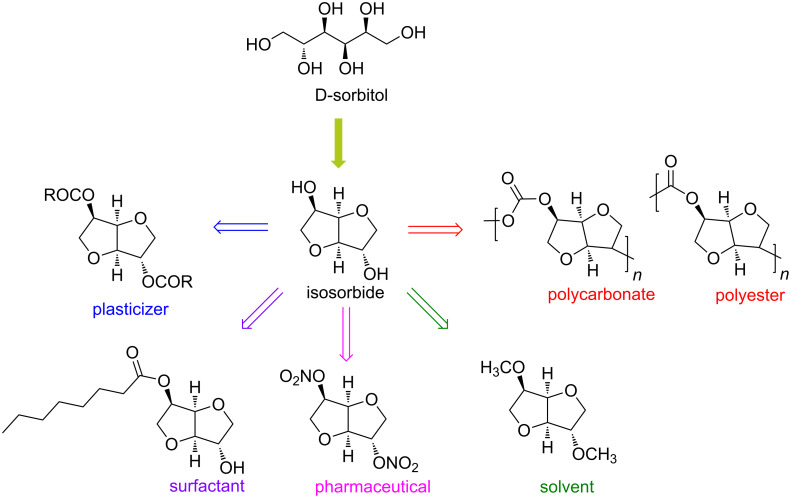
Chemical structure of isosorbide and its epimers isomannide and isoidide.

**Scheme 1:**
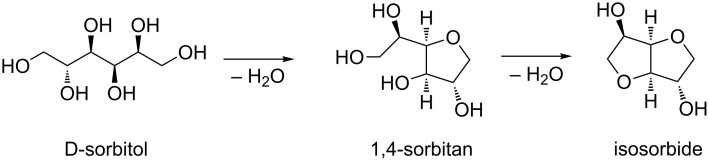
Conversion of D-sorbitol to isosorbide via twofold dehydration reaction.

The reason of such interest relies on the fact that D-sorbitol has all the characteristics of a typical bio-based platform chemical in terms of sustainability, applications and market value. In fact, dehydration of D-sorbitol (Scheme 1) produces anhydro sugar alcohols, including sorbitan (mono-anhydrosorbitol) and isosorbide (dianhydrosorbitol). Both these products have achieved commercial importance and can be used to synthesize numerous intermediates of industrial interest (Figure 2). Selected examples include isosorbide nitrate derivatives, well-known vasodilator drugs for treatment of heart-related deseases [4–5]; isosorbide alkyl esters, bio-based plasticizers [6–10] and short-chain aliphatic isosorbide ethers that have recently found application as coalescent for paints (Figure 2) [11–14].

The isosorbide moiety has also been incorporated in several bio-based polymers, i.e., poly(ethylene-*co*-isosorbide)terephthalate (PEIT), poly(isosorbide oxalate) and poly(isosorbide carbonate) [15–18] such as DURABIO^®^ and PLANEXT^®^.

Furthermore dimethyl isosorbide (DMI; bp 235 °C) [19], has found applications as potential substitute of high-boiling solvents (DMSO, DMF) and long chain aliphatic ester derivatives of isosorbide (mono- and disubstituted) have been investigated as surfactants [20].

However, it should be pointed out that, despite D-sorbitol and isosorbide are renewable materials, their derivatizations do not always follow the green chemistry principles. In this prospect, the present work is focussed on the reactivity of D-sorbitol and isosorbide with the green reagent and solvent dimethyl carbonate (DMC).

Dimethyl carbonate, the simplest among the dialkyl carbonate (DAC) family, is nowadays produced by a clean and halogen-free process [21–23]. This compound has been extensively employed as green substitute of highly toxic phosgene in carboxymethylation reactions and methyl halides or other noxious methylating agents in methylation reactions [24–35].

The reactions between the bio-based chemicals D-sorbitol or isosorbide and DMC, are very appealing as they encompass the preparation, as well as the transformation of a renewable resource into industrially relevant products via a chlorine-free and green approach.

### Synthesis of isosorbide via dimethyl carbonate

The current research on the preparation of D-sorbitol is mainly focussed on direct hydrolytic hydrogenation of cellulose [36–39] via a two-step reaction:

1. Conversion of cellulose into glucose by hydrolysis.
2. Hydrogenation of glucose to D-sorbitol.

An appropriate catalyst for this process should provide both acid sites (hydrolysis) and metallic sites (hydrogenation). Thus, several bifunctional catalytic systems have been investigated [40–44]. The use of ionic liquids as reaction media has been also taken into consideration, although their use is limited by solubility problems and environmental concerns [45–48].

Despite the continuous research on its direct preparation from cellulose, D-sorbitol is nowadays synthesized on industrial scale by hydrogenation of glucose via biotechnological and chemocatalytic depolymerization of polysaccharides. The conversion of D-sorbitol into isosorbide via sorbitan is then usually performed by a twofold dehydration reaction using different types of catalysts (Scheme 1) [49–60].

In 1968 Fleche and co-workers reported the first synthesis of isosorbide from D-sorbitol using sulfuric acid as catalyst [61–62]. The reaction was performed at 400 K in a batch reactor. This process results in good yields (ca 70%), but it also poses some issues such as difficult separation of isosorbide from the reaction mixture and the use of a large amount of sulfuric acid. As a result current research on new synthetic approaches for the cyclic sugar isosorbide has been focussed on less toxic and easy to recover heterogeneous acidic catalysts. In particular, mixed oxides [49], phosphated or sulfated oxides [50–56], sulfonic resins [57–59] and bimetallic catalysts [60] have been investigated.

Extensive work has also been conducted on the use of zeolites, which compared to the above mentioned catalysts, have the advantage to be thermal stable and possess tuneable properties. However, zeolites are not very efficient catalysts for the dehydration of D-sorbitol as isosorbide yields usually range between 40 to 60% [57,63–64]. Furthermore they also require high temperature, i.e., 430–533 K.

Recently Fukuoka and co-workers reported a new efficent Hβ zeolite with a high Si/Al ratio (up to 75) that showed an improved activity and allowed dehydration of D-sorbitol into isosorbide in 76% yield at 400 K (127 °C) [65]. The Hβ zeolite can also be reused up to five times before losing its activity as catalyst.

Despite this methodology being one of the most promising so far reported, it still requires the separation and purification of isosorbide from the reaction mixture. In this view, a different synthetic approach to isosorbide employs the versatile, green reagent and solvent dimethyl carbonate (DMC) as dehydrating agent.

The reaction between D-sorbitol and DMC performed in the presence of a base at reflux temperature (90 °C) leads to the high yielding formation of isosorbide (Table 2). The advantage of this synthesis is that the reagents are commercially available and isosorbide can be easily recovered by filtration of the excess of base and removal of the solvent which can be eventually reused.

**Table 2:** Synthesis of isosorbide by DMC chemistry.^a^

entry	Solvent	Cat./base (equiv)	DMC (equiv)	Time (h)	Isosorbide % (% isolated yield)

1	None	NaOMe (2.0)	20	8	16
2	MeOH	NaOMe (2.0)	4	8	80 (64)
3	MeOH	NaOMe (4.0)	8	8	98 (76)

4	MeOH	DBU (1.0)	8	7	100 (98)
5	MeOH	DBU (0.25)	8	7	100 (98)
6	MeOH	DBU (0.05)	8	24	100 (98)

^a^Reaction conditions: D-Sorbitol 2 g (1 equiv); reflux temperature; conversion of the starting material was in all cases quantitative.

A first set of experiments was conducted at 90 °C and atmospheric pressure using an excess of strong base, i.e., sodium methoxide (entries 1–3, Table 2). In particular, when the reaction was performed in the presence of 2 equiv of sodium methoxide, isosorbide was formed only in modest yield (entry 1, Table 2).

**Scheme 2:**
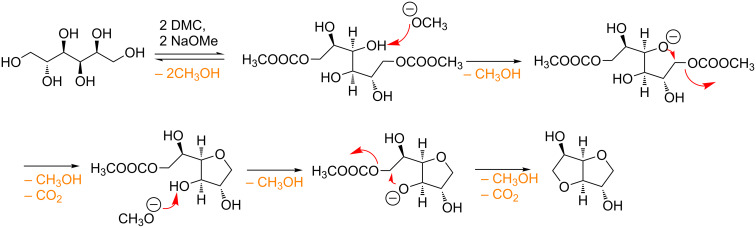
Possible reaction mechanism for the conversion of D-sorbitol to isosorbide.

The main issue of this procedure was that isosorbide, once formed, can further react with DMC leading to the formation of its methoxycarbonyl and methyl derivatives [35]. However, when methanol was used as a solvent (entries 2–3, Table 2), the numerous equilibria that affect the formation of the product can be efficiently shifted towards isosorbide preventing any further reactions (Scheme 2). Best results were achieved when an excess of NaOMe was employed (entry 3, Table 2). The necessity of an excess of base might be ascribed to the complexity of this one-pot double cyclisation reaction that requires 2 equiv of base for each tetrahydrofuran formed.

The reaction mechanism is quite complex (Scheme 2) since it encompasses two carboxymethylation reactions (via B_Ac_2) followed by two intramolecular cyclisations (via B_Al_2).

In order to avoid the use of excess base, several alternative catalysts and bases have been taken into consideration. Recently it has been reported that 1,5-diazabiciclo(5.4.0)undec-5-ene (DBU) can be used in stoichiometric amounts for the efficient synthesis of isosorbide via DMC chemistry (entry 4, Table 2) [66]. Under these reaction conditions, isosorbide was obtained in pure form by filtration on a silica pad and evaporation of the DMC. Even when the amount of DBU was reduced to 5 mol % (entries 4–6, Table 2) the cyclic sugar was still formed in quantitative yield. It is also noteworthy that, although the catalyst employed is homogenous, the amount of DBU used was, in the latter case (entry 6, Table 2) only 2.5 mol % for each tetrahydrofuranic cycle. The same synthetic approach can be also employed for the cyclisation of D-mannitol.

The synthesis of isosorbide via DMC chemistry takes advantage of the enhanced reactivity of DMC in the presence of the nitrogen bicyclic base DBU. It has been, in fact, reported that organic carbonates are activated by DBU via formation of an *N*-alkoxycarbonyl DBU derivative [67–71]. However, in this case study, DBU most probably promotes the formation of the methoxycarbonyl reaction intermediate, as well as the intramolecular cyclisation reaction (B_Al_2 mechanism).

It is also noteworthy that in general alkylation reactions promoted by DMC chemistry are conducted at temperatures above 150 °C [24–35], but in this case study the intramolecular cyclisation step leading to isosorbide, which is an alkylation reaction (Scheme 2), takes place at the DMC refluxing temperature (90 °C).

To explain this result, computational investigations were conducted on a model compound. The collected results demonstrated that the cyclisation reaction leading to the 5-membered ring is a preferred pathway compared to other possible ones (7-membered ring closure, alcoholate attacks onto DMC) due to a big entropic effect [35].

### Reactivity of isosorbide with dimethyl carbonate

One of the most investigated research fields for the sustainable platform chemical isosorbide is the synthesis of bio-based polymers (Figure 1). In fact, isosorbide has been extensively employed for the preparation of polyesters, polyurethanes and polycarbonates [72–81]. Isosorbide is also considered as a possible candidate to replace petroleum-derived and toxic bisphenol A in polycarbonate preparation. In this view, the main issue that limits the exploitation of this compound is its lower acidity. To overcome this problem, polycarbonates incorporating an isosorbide moiety have been synthesized via a chlorine-based approach, i.e., employing phosgene or its derivatives [82–83].

**Scheme 3:**
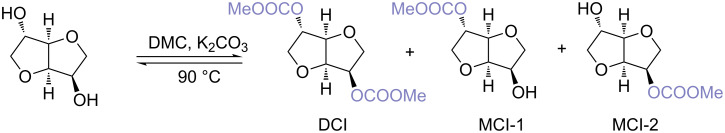
Methoxycarbonylation of isosorbide via DMC chemistry.

**Scheme 4:**
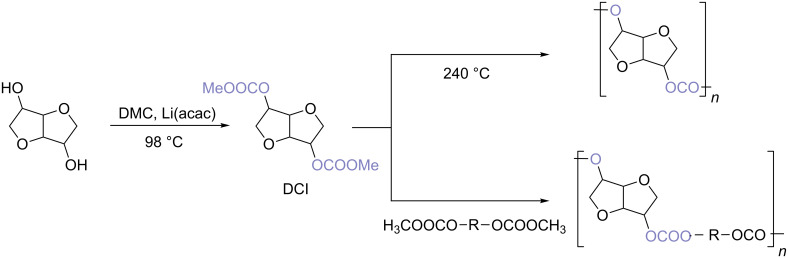
Isosorbide homo- and co-polycarbonate via melt polycondensation.

On another hand, a greener synthetic methodology to bio-based polymers is to first synthesize a more reactive derivative of isosorbide and then perform the polycondensation reaction (Scheme 4). In this prospect a good candidate is the dicarboxymethyl isosorbide (DCI). In fact, methoxycarbonylation of isosorbide via DMC chemistry is a relative simple reaction that has been extensively investigated (B_Ac_2 mechanism according to Scheme 3).

Data reported in the literature show that carboxymethylation of isosorbide can be achieved by reacting isosorbide with an excess of DMC at refluxing temperature in the presence of potassium carbonate (Table 3) [84]. Under these conditions, due to the presence of four chiral centres in the isosorbide backbone, three products can be formed, the wanted dicarboxymethyl carbonate (DCI) and two monocarboxymethyl carbonates MCI-1 and MCI-2 (Scheme 3).

**Table 3:** Synthesis of dicarboxymethyl isosorbide (DCI) by DMC chemistry.^a^

#	K_2_CO_3_	Selectivity (%)		

	(equiv)	MCI-1	MCI-2	DC

1^b^	1.00	37	9	54
2	1.00	10	5	85
3	0.50	11	4	85
4	0.10	8	2	90

^a^Reaction conditions: isosorbide DMC 1:30 equiv; temperature 90 °C; reaction time 6 h. All the reactions have been conducted under anhydrous conditions. Conversion was always quantitative. ^b^The reaction has not been conducted under anhydrous conditions.

When isosorbide was reacted with an excess of DMC (30 equiv) in the presence of a stoichiometric amount of K_2_CO_3_ (1 equiv), a quantitative conversion of the substrate was observed, but the selectivity toward DCI was just moderate. Monocarboxymethyl derivatives MCI-1 and MCI-2 were still present in the reaction mixture (entry 1, Table 3).

However, repeating the reaction under anhydrous conditions, the selectivity towards DCI increased to 85% (entry 2, Table 3). Most probably, even a small amount of water can affect the outcome of the reaction as it can hydrolyse the DMC molecule into CO_2_ and methanol. The latter, once formed, shifts the reaction equilibrium towards the reagent and the monocarboxymethyl derivatives. When the reaction is performed under anhydrous conditions, the amount of potassium carbonate can be decreased up to 10 mol % (entries 3 and 4, Table 3) without affecting the reaction outcome.

Recently DCI has been also prepared via DMC chemistry in the presence of lithium acetylacetonate (Li(acac)) as catalyst [85]. Dicarboxymethyl isosorbide, once formed, has been directly converted into either homo- or co-polycarbonate via an easy straight-forward procedure (Scheme 4).

In the case of homopolymer preparation, DCI was synthesised by reacting isosorbide, DMC and Li(acac) at 98 °C. The polycondensation was then achieved employing an high vacuum and increasing the temperature to 240 °C. The so-formed poly(isosorbide carbonate) had a molecular weight (*M*_n_) of 28,800 g/mol. The conversion of isosorbide was almost quantitative (95.2%).

Similarly poly(aliphaticdiol-*co*-isosorbide carbonate)s were prepared via melt polycondensation of DMC with isosorbide and several aliphatic diols employing Li(acac) and the TiO_2_/SiO_2_-based catalyst (Scheme 4) [85].

High-molecular-weight (*M*_w_ = 32,600) and optically clear isosorbide-based polycarbonates were also reported by Shin and co-workers [86]. However, in this case, the polymerisation reaction was conducted using diphenyl carbonate in the presence of a catalytic amount of cesium carbonate.

Another interesting isosorbide derivative is dimethyl isosorbide (DMI) that has potential application as green solvent substitute of high boiling polar solvents. Recently DMI has also appeared as component in the formulation of deodorants [87].

**Scheme 5:**
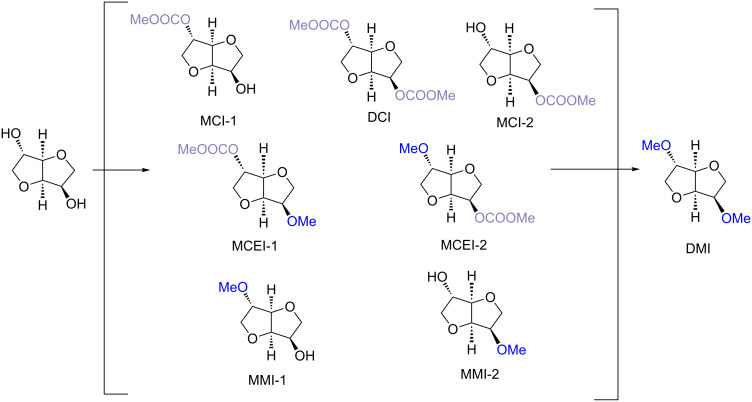
Synthesis of DMI via DMC chemistry.

Methylation of isosorbide has been investigated both at reflux and in autoclave conditions via DMC chemistry. It should be mentioned that generally methylation of secondary alcohols via DMC chemistry requires high temperatures (>150 °C) and was never obtained in high yield due to the formation of elimination products [88]. However, isosorbide, which incorporates in its backbone secondary hydroxy groups, was quantitatively methylated at the reflux temperature of DMC (90 °C) in the presence of a base (Table 3) [19]. This is particularly significant since the reaction of isosorbide with DMC (Scheme 5) can lead to the formation of numerous compounds such as: carboxymethyl derivates (MCI-1, MCI-2, DC), carboxymethyl methyl derivates (MCEI-1, MCEI-2) and methyl derivates (MMI-1, MMI-2 and DMI).

As reported in Table 4 performing the methylation reaction at the reflux temperature of DMC in the presence of a strong base (stoichiometric amount) resulted in a moderate yield of DMI (entries 1 and 2, Table 4). Quantitative conversion of isosorbide into DMI was obtained only using an excess of sodium methoxide (entry 3; Table 4).

**Table 4:** Synthesis of dimethyl isosorbide (DMI) by DMC chemistry.^a^

entry	Base	Temp	Selectivity (%)^b^				

	(equiv)	(°C)	DMI	MMI-1	MMI-2	MCEI-1	MCEI-2

1	*t*-BuOK (1.5)	90	40	2	2	37	18
2	NaOMe (1.5)	90	26	11	6	30	12
3	NaOMe (3.0)	90	100	0	0	0	0

4^c^	K_2_CO_3_ (1.0)	200	57	4	7	29	0
5^c^	*t*-BuOK (1.0)	200	55	5	6	34	0
6^c^	KW2000^d^	180	83	1	3	12	0
7^c^	KW2000^d^	200	86	0	2	12	0

^a^Reaction conditions: Isosorbide DMC 1:50 equiv; Reaction time 20 h; Conversion 100%. ^b^Carboxymethyl derivatives MCI-1, MCI-2 and DC have been detected only in traces. ^c^Reaction conducted in an autoclave under pressure. ^d^Hydrotalcite was calcinated at 400 °C overnight prior its use.

In order to optimize the reaction conditions and reduce the amount of catalyst, the methylation of isosorbide was also conducted in an autoclave at higher temperature in the presence of a base. Using weak base K_2_CO_3_ in stoichiometric amount at 200 °C already resulted in a selectivity towards DMI of ca. 57%. Comparable results were achieved by using a stronger base, i.e., *t*-BuOK, (entry 5, Table 4).

However, when hydrotalcite KW2000 (Mg_0.7_Al_0.3_O_1.15_), a catalyst that incorporates both acidic and basic sites, was used (1:1 w/w ratio) DMI formed in good yield (86%) (entries 6 and 7, Table 4). Hydrotalcite has the advantage to be heterogeneous, thus it can be eventually recycled. The reaction mechanism involving hydrotalcite is not yet fully understood, most probably the acidic sites activates the DMC molecule and at the same time the basic sites activate the substrate.

**Scheme 6:**
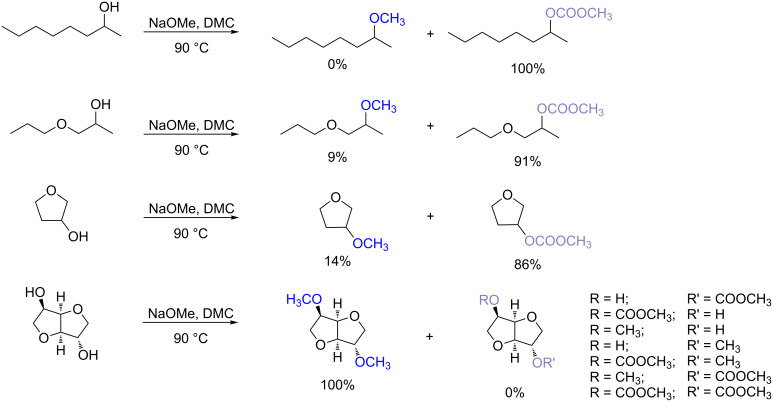
Comparison of the reactivity of isosorbide with other secondary alcohols in methylation reaction. Reaction conditions: Isosorbide DMC 1:50 equiv; reaction time 20 h; 90 °C.

Interestingly, isosorbide peculiar backbone seems to play a very important role in the methylation reaction via DMC chemistry. In fact when the methylation via DMC reaction was performed on other secondary alcohols in the best found conditions at the reflux temperature of DMC, methyl derivatives were either not observed or formed in small amount (Scheme 6). In particular, 2-octanol gave only the carboxymethyl derivative, meanwhile the methyl derivatives of propylene glycol propyl ether and 3-hydroxytetrahydrofuran formed only in scarce amount. Among the substrates investigated, isosorbide was the only one leading to almost quantitative methylation confirming the influence of its peculiar backbone on the reactivity of this compound.

**Figure 3:**
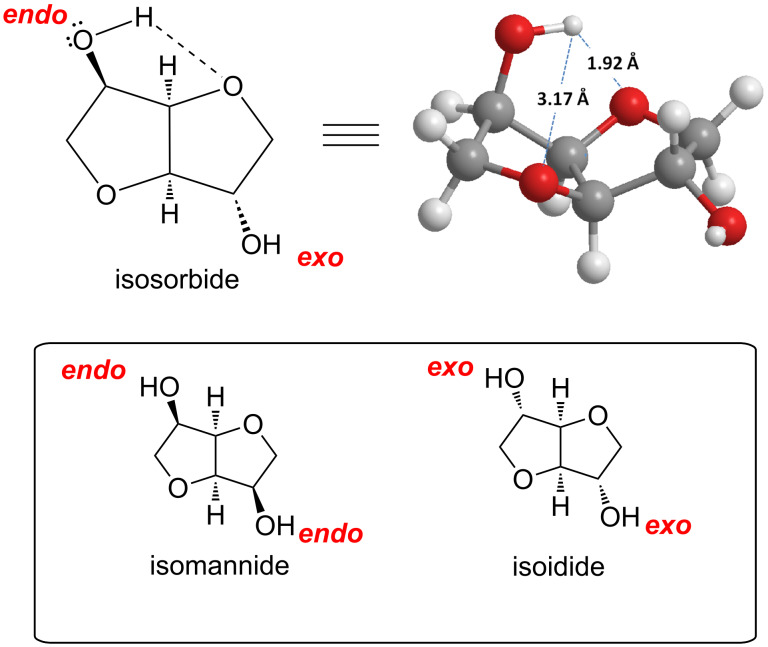
Chemical structure of isosorbide and its epimers isomannide and isoidide.

In fact, the growing interest in isosorbide is justify not only by its bio-based nature and industrial applications, but also by its high reactivity and peculiar molecular structure [89]. Isosorbide has an open-book V-shaped configuration formed by two *cis*-connected tetrahydrofuran rings with an opening angle of 120°. The four oxygen atoms incorporated in the structure are in β-position to each other [61–62]. The secondary hydroxy moiety in the 2-position directed toward the V-shaped cavity is labelled as *endo*, meanwhile the one in the 5-position pointing outside of the sugar cavity is indicated as *exo* (Figure 3).

The configuration of the two hydroxy groups has been shown to influence the reactivity of isosorbide. In fact, its epimers, isoidide and isomannide, that incorporate only *exo* or *end*o hydroxy groups, have different physical/chemical properties, as well as diverse reactivity. Thus, the easy methylation of isosorbide is most probably due to the unique V-shaped structure of isosorbide in combination with the presence of four oxygen atoms all in β position to each other that enhance the nucleophilicity of the hydroxy groups.

## Conclusion

Among the top chemical opportunities from biorefinery carbohydrates D-sorbitol is a platform chemical of considerable interest that has led to intensive research in the last years especially as the parent alcohol of isosorbide. The latter is also a platform chemical with applications in pharmaceuticals, detergents, fuel additives, monomers and building blocks for new polymers and functional materials and new high boiling organic solvents. Conversion of D-sorbitol into isosorbide and its consequent transformation into valuable derivatives is under intense investigation.

In this review, we have focussed on the reactivity of D-sorbitol and isosorbide with the green reagent and solvent DMC as a relevant example of green and halogen-free chemistry. It has been, in fact, reported that dehydration of D-sorbitol can be efficiently conducted using DMC used as dehydrating agent in the presence of a catalytic amount of the homogenous catalyst DBU under mild conditions. Compared to the other synthetic pathways reported in the literature, the DMC based synthetic approach is a “chlorine-free”, one-pot and environmental friendly method that does not require any time consuming purification technique and allowed isolation of a very pure crystalline product using commercially available reagents. To the best to our knowledge, this synthetic approach is the one resulting in the highest isolated yield.

Dicarboxymethyl isosorbide is also an intermediate of great interest in view of its application as monomer for homo- and co-polycarbonates incorporating the isosorbide subunit.

In this prospect, carboxymethylation of isosorbide can be efficiently carried out via DMC chemistry via a B_Ac_2 mechanism employing a catalytic amount of K_2_CO_3_ at reflux conditions in anhydrous conditions.

Li(acac) has also been reported as efficient and selective catalyst that was efficiently used for carboxymethylation reaction of isosorbide and its consequent polymerization reaction to achieve bio-based polymers.

Another interesting derivate of isosorbide is dimethyl isosorbide that has potential in applications as green high boiling bio-based solvent. In this case, DMC was efficiently used as methylating agent of isosorbide at its reflux temperature (90 °C) in the presence of an excess of base. This result was ascribed to the neighbouring effect of the oxygen situated all in β-position to each other that most probably enhances the nucleophilicity of the corresponding hydroxy group.

Furthermore the amphoteric catalyst hydrotalcite was extremely efficient in the synthesis of DMI when tested in an autoclave at higher temperature and has the additional advantage that it can be recycled.

It is thus noteworthy that the reactions involving bio-based platform compounds D-sorbitol and isosorbide with green reagent and solvent DMC encompass free-halogen chemistry to achieve industrially relevant products that might substitute fossil-based compounds and that are a poignant example of innovation at molecular level that nicely combines green chemistry reactions with biorefinery of carbohydrates.
